# Transport and Survival of Marine Tracer Phages in Topsoil at Field Conditions

**DOI:** 10.1021/acs.est.5c12252

**Published:** 2025-12-23

**Authors:** Konstanze Hild, Nimo Kwarkye, Chen Huang, Hauke Harms, Antonis Chatzinotas, Thomas Ritschel, Kai U. Totsche, Lukas Y. Wick

**Affiliations:** † Department of Applied Microbial Ecology, 28342 Helmholtz Centre for Environmental Research - UFZ, Permoserstraße 15, 04318 Leipzig, Germany; ‡ Department of Hydrogeology, 9378 Friedrich-Schiller University Jena, Burgweg 11, 07749 Jena, Germany; § Department of Biogeochemical Processes, 28300 Max Planck Institute for Biogeochemistry, Hans-Knöll-Straße 10, 07745 Jena, Germany; ∥ Institute of Biology, Leipzig University, Talstraße 33, 04103 Leipzig, Germany; ⊥ German Centre for Integrative Biodiversity Research (iDiv) Halle-Jena-Leipzig, Puschstraße 4, 04103 Leipzig, Germany; # Cluster of Excellence Balance of the Microverse, Friedrich-Schiller-University Jena, Fürstengraben 1, 07743 Jena, Germany

**Keywords:** soil, (bio)colloidal tracer, pasture, forest, infectivity, viruses

## Abstract

Phages are ubiquitous in soil, shaping microbial diversity and nutrient cycling. Phage replication requires maintaining infectivity and finding the right host. Yet, there are limited data on phage persistence and transport in soil under field conditions. The potential presence of hosts enabling phage replication impedes the assessment of the mobility of autochthonous phages in soils. In lysimeters installed in forest and pasture topsoil, we elucidated the transport of the tailed marine *Pseudoalteromonas* phage HS2 in comparison to deuterium. Transport of infectious phages as well as numbers of tracer phage genomes and tracer capsid-bound genomes were quantified to account for phage retention and inactivation. Phages were transported up to 4 times faster than the simultaneously applied deuterium tracer, which was attributed to pore size exclusion. Retention in immobile regions and remobilization during precipitation caused pronounced tailing in tracer breakthroughs. High phage survival in pasture soil resulted in mass recoveries of infectious phages that were up to 6 times higher than those in forest soil. However, long-term observations showed that the infectivity was also preserved in forest soil, enabling event-driven remobilization. This remobilization underscores the importance of distinguishing between phage retention and inactivation, which is crucial for accurately predicting phage transport dynamics and their ecological impact in terrestrial environments.

## Graphical abstract

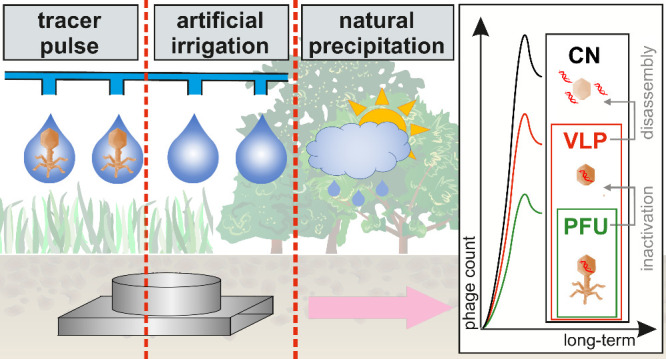

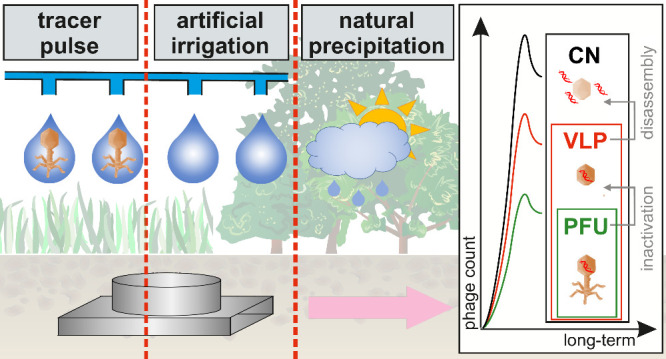

## Introduction

Soils are among the largest viral reservoirs on Earth, typically harboring 10^7^ to 10^10^ viruses per gram of soil.^1,2^ The soil virome predominantly contains tailed bacteriophages (phages),^3,4^ i.e., viruses that infect bacteria, thereby maintaining and driving microbial diversity^5^ and shaping nutrient cycling.^6^ As microbial hosts in heterogeneous soils are often spatially dispersed, the ecological function of phages depends decisively on their ability to find an appropriate host before losing their infectivity in a demanding environment. Phages lack motility and must therefore be transported passively, either suspended within fluids, or attached to mobile soil components^7,8^ or to soil microorganisms like fungi and nonhost bacteria.^9^ Yet, pathways of preferential flow to deeper subsurface layers^10^ do not necessarily coincide with the habitats of the host. Moreover, phage retention in immobile regions^11,12^ may prolong the phage–host encounter, while the adsorption of phages to biogeochemical interfaces^13,14^ or fungal mycelia^15^ also impedes their transport. Despite their low thickness, topsoils are a critical part of phage migration due to the highest microbiological activity and diversity, as well as a high content of organic matter and pedogenic clay minerals. Along with time,^16^ environmental factors such as pH,^17,18^ ionic strength,^19^ temperature,^20,14^ saturation conditions,^21^ and mechanical stress^22,23^ can cause a loss of infectivity. Consequently, an assessment of the ecological relevance of phages and their potential to spread in the subsurface requires knowledge of their ability to maintain infectivity while being transported through the subsurface, particularly soils.

Assessing the persistence of phages in natural soils is challenging due to the inaccessibility of subsurface environments and the potential presence of host bacteria. In prior laboratory column and field experiments, nontailed phages were applied to soil or other porous media (e.g., sand, karst), and the removal of infective phages was quantified based on plaque-forming units (PFUs).^24–29^ However, nontailed phages have been used as proxies for human viruses and sewage contaminants,^30^ but they may not accurately reflect the transport mechanisms of a significant proportion of soil phages, which are mainly tailed.^3,4^ The tail is particularly susceptible to mechanical stress during transport, which can potentially lead to breakage and the loss of infectivity.^22^ This is not accounted for when using nontailed phages, which can result in an inaccurate representation of phage infectivity in soil. In addition, if only PFUs are quantified in phage transport experiments, then any infectivity loss may be misinterpreted as retention during transport. However, these processes can be differentiated by comparing the number of virus-like particles (VLPs), which represent the infective and noninfective phages, with that of PFUs, which represent only infective phages.^22,31^ Such differentiation can, e.g., be achieved by comparing the fate of native and labeled phages which was previously done in batch experiments.^32^ However, qPCR can be used, as done in previous laboratory transport experiments,^31^ to additionally assess capsid disassembly by comparing the copy number of the tracer phage genome in total DNA (tCN) with its copy number in DNA extracted only from intact VLPs (i.e., vlpCN). Consequently, an investigation into the viability of phages during transport in soil necessitates long-term studies with phages that represent the most commonly found morphology in soil as well as an analytical approach that differentiates between infectivity loss and PFU retention with subsequent potential remobilization.

Tailed marine phages have been applied to trace ground and surface water flows,^33–35^ and their potential to mimic transport of tailed phages in soil has already been shown in laboratory columns.^22,31^ However, the long-term transport of marine tracer phages has not been tested under soil field conditions so far. Their natural absence in soil facilitates the concise detection and tracing of their transport. Moreover, the application of marine tracer phages is unlikely to influence soil ecology as their natural marine host bacteria are assumed to be absent. Vice versa, the absence of their host further impedes any replication during transport. This allows the characterization of tracer phage transport and survival only. Marine phages are thus promising tracers for investigating phage mobility and the loss or maintenance of infectivity. While previous studies revealed the response of phage migration to different physicochemical boundary conditions, their transport under transient natural conditions has not yet been explored.

We applied and monitored the transport of the marine *Pseudoalteromonas* phage HS2, i.e., a phage reflecting the morphology of the majority of soil phages (siphovirus),^3^ under field conditions and atmospheric forcing using lysimeters constructed on two prototypical land cover types (pasture and forest). Pasture soils, which are influenced by grazing and agricultural practices, differ from forest soils in soil properties^36,37^ and microbial compositions,^38^ potentially resulting in different phage movement and survival. Tracer phage PFUs, tCN and vlpCN were quantified in the lysimeter seepage. In contrast to previous studies relying on only PFU counts, our study allowed us to distinguish between phage transport and loss of particle integrity, including loss of infectivity, during transport in undisturbed soil. Phage transport was compared with that of deuterated water as reference for fluid flow.^39^ This represents the first long-term time series (i.e., one year) of tailed tracer phage transport under conditions of natural fluid flow. Thereby, we demonstrate the long-term survival of tailed phages and their potential remobilization in natural soils.

## Materials and Methods

### Preparation and Quantification of Marine Tracer Phages

The marine *Pseudoalteromonas* phage HS2 was propagated using cultures of its host bacterium, *Pseudoalteromonas* H13–15, in liquid medium (PZ-Zobell), following the method described by You et al.^31^ Phages were then purified by centrifugation (8000 × g, 10 min), and the supernatant was filtered (pore size 0.22 μm, diameter 25 mm, CA-filters, LABSOLUTE, Th. Geyer GmbH & Co. KG, Renningen, Germany) and concentrated by ultracentrifugation (22000 rpm, 2 h, 4 °C). The redissolved pellet was then filtered again to ensure the absence of bacteria as verified by streak plating.

PFUs, which were used as a proxy for infectious phages, were quantified after preparing a dilution series of seepage water and applying 5 μL droplets of each dilution to a double-layer plaque assay, with a limit of quantification (LOQ) of 600 PFU/mL.^40^

To quantify the copy numbers of the tracer phage genome, seepage water was diluted in SM buffer, vortexed for 20 min, and then centrifuged to remove soil particles (3000 × g, 30 min) following Narr et al.^2^ Two DNA extractions were done with the supernatant using the QIAamp Viral RNA Mini Kit (QIAGEN, Hilden, Germany); one extraction was performed directly to quantify the total copy number (tCN) of the marine tracer phages. The other extraction was done after DNase I (Ambion, Thermo Fisher Scientific, Waltham, MA, USA) treatment to remove any ambient DNA present in the samples due to capsid disassembly, and to quantify the copy number in intact VLPs (vlpCN),^31^ providing LOQs of 2.9 × 10^5^ tCN/mL and 2.6 × 10^5^ vlpCN/mL. Quantification of vlpCN and tCN was performed by qPCR following the protocol of You et al.^31^ The proportion of tracer phages with intact capsids was estimated by comparing vlpCN to tCN.^31^ The ratio of PFU to vlpCN was used to assess the specific infectivity^22^ of the tracer phages after transport.

### Quantification of Deuterium Concentration in Lysimeter Seepage

Each collected seepage sample was placed in a 1.5 mL brown glass vial and stored at 4 °C until further deuterium analysis. The deuterium signal was analyzed using high-temperature conversion isotope ratio mass spectrometry (Delta V Plus, Thermo Scientific, Waltham, USA). Samples were heated above 1350 °C in a glassy carbon reactor,^41^ and the resulting H_2_ was analyzed. Measurement results were first reported as δ^2^H (‰), indicating the relative difference in the ^2^H/H ratio of the sample versus the ratio in standard, i.e., VSMOW (Vienna Mean Ocean Water) and SLAP (Standard Light Antarctic Precipitation), normalized by the VSMOW-SLAP ratio.

To calculate the mass recovery of deuterium during the tracer application period, the measured δ^2^H (‰) values were converted to the atom fraction (*X*) to avoid nonlinearity of the δ expression. The δ^2^H was first converted to the D/H ratio (*R*), where the standard *R*_VSMOW‑SLAP_ = 0.00015575.

1 $$R_{\mathrm{sample}}=R_{\mathrm{VSMOW}}\times \left(1+\frac{\delta^{2}H}{1000}\right)$$

The atom fraction of deuterium (*X*_D_) was then calculated as

2 $$X_{D}=\frac{R}{1+R}$$

The excess deuterium atom fraction in each sample was computed by subtracting the deuterium atom fraction from the background samples. Based on the collected sample volume, the moles of H atoms and excess moles of D in each sample were calculated, under the assumption that the water density is 1 g/mL and the molar mass of water is 18.015 g/mol. Deuterium concentration as mg/L can be further calculated as atomic mass ≈ 2.014 g/mol. The recovery of deuterium (D_r_) in the sample was ultimately calculated by summing all the excess mass of D in each sample relative to the mass of D applied in the tracer solution. Estimated D_r_ in each lysimeter was assumed to represent the fraction of tracer solution reaching the lysimeter. As such, the total amount of tracers reaching the lysimeter was calculated as the product of the amount of tracer in solution and the D_r_ of the corresponding lysimeter.

### Site Description and Lysimeter Setup

The study area is located in Hainich National Park, NW Thuringia, central Germany. The climate in the region is characterized by warm summers, a mean annual air temperature of about 9.5 °C, and an average annual precipitation of about 600 mm. The predominant soil types in the study area are Cambisols and Luvisols, which developed from the weathering of limestone and mudstone alterations, the primary bedrock material of the site.^8^

The transport experiments were conducted using four lysimeters^8^ constructed as part of the Hainich Critical Zone Exploratory (Collaborative Research Center CRC 1076 AquaDiva).^42^ The lysimeters were chosen to represent two prototypical land uses (pasture and forest) and were located close to each other within a range of less than 100 m. Hence, soil development was driven by the same climatic conditions (precipitation, temperature), host rock, and topographic relief, and differences in soil properties were solely caused by land use, the associated vegetation, and input of litter. Specifically, two lysimeters were located in pasture vegetation (hereafter referred to as P1 and P2) and two in forest vegetation (hereafter referred to as F1 and F2). A comprehensive description of the lysimeters, including the construction methodology and soil parameters such as bulk density and porosity, is provided by Lehmann et al.^8^ and the Supporting Information (SI) therein. In brief, tension-controlled lysimeters were installed at the topsoil–subsoil boundary as assessed in Lehman et al.^8^ resulting in a different depth of the lysimeters (30 cm below the pasture site surface and 23 cm below the forest site surface),^8^ collecting seepage from the undisturbed topsoil in the pasture and forest sites, respectively. The lysimeters were installed horizontally with minimal disturbance to preserve the natural soil structure. All lysimeters were equipped with porous plates with a maximum pore size of 119 μm, enabling the collection of both dissolved and colloidal components of the mobile inventory.^8^

### Tracer Application

Based on previous work by Ghanem et al.^22,43^ and You et al.,^31^ we included the marine *Pseudoalteromonas* phage HS2 into the set of tracers applied in a joint sequential multitracer experiment, which also employed deuterated water, fluorescein, and poly­(ethylene glycol)^44,45^ (Table S2). We applied 3.1 × 10^10^ PFU/mL, which was equal to 1.1 × 10^11^ vlpCN/mL or 4.8 × 10^11^ tCN/mL. The interaction of phages with other tracers was negligible, and no effect on the infectivity was observed (Figure S2). The tracers were applied in late April 2023, coinciding with periods of optimal temperature conditions that precluded the possibility of overnight soil freezing that might compromise phage infectivity. A sprinkler-irrigation unit design was adopted^46^ and adjusted to meet the requirements of chemical inertness toward the tracers/phages and spatial uniformity of the tracer solution over the lysimeters. This resulted in natural flow conditions induced by precipitation and unsaturated water flow throughout the experimental period. Unless otherwise specified, 0.59 mM sodium chloride (Roth, Karlsruhe, Germany) was used as the background solution throughout the tracer application. The lysimeters were irrigated with 15 L per lysimeter for three h per day, which simulated a heavy rain event with a rate of 20 mm/h. The irrigation used the following scheme with three phases (Figure S1):

I. Days 1 and 2: preconditioning of the lysimeters with background solution.
II. Day 3: irrigation with tracer solution.
III. Days 4–6: irrigation with background solution.

Details on the tracer application period and the quantity applied to each lysimeter are presented in Tables S3–S6. Four to five samples were taken at each lysimeter during the irrigation period and whenever possible, such as after precipitation events following the application period. A singular occurrence of strong outliers in the deuterium signal and sample volume showed that two samples, taken 21 h (P1) and 22.5 h (P2) after tracer application from pasture lysimeters, have most likely been mislabeled; the corresponding data points were therefore exchanged. After forced irrigation, we continued sampling for more than eight months whenever seepage occurred due to precipitation events.

## Data Acquisition and Analysis

### Tracer Breakthrough Assessment

As natural precipitation was absent during the irrigation period, we considered the timespan from the beginning of the irrigation to 3 days after irrigation as the period of irrigation-induced infiltration and later seepage as driven by natural precipitation. The recovered phage mass (i.e., recovered tracer PFU, vlpCN or tCN) was calculated as the sum of all phage mass in collected samples during the irrigation-induced period. Recovered phage mass in each lysimeter was normalized to the product of total phage mass in tracer solution and D_r_ of corresponding lysimeter to obtain the normalized mass recovery (MR). This facilitated distinguishing between losses due to transport and phage interactions/retention in soil. Additionally, relative tracer mass transports were estimated and compared between deuterium and phages, as well as between sites. Specifically, the time taken to transport 10%, 25%, 50%, 75%, and 90% of the total tracer mass during the irrigation-induced period was determined. Due to the low thickness of topsoils, a continuum-scale transport behavior as required for common transport models cannot be presumed. Hence, using those models to quantitatively assess continuum-scale phage transport parameters, e.g., phage retention, inactivation, interaction rates, and storage in immobile pore regions^47^ may lead to a misrepresentation of the underlying transport processes.^48^ Therefore, nonparametric measures of breakthrough curves, like the average tracer arrival time and average transport velocity, were estimated to compare breakthrough curves as described in Koestel et al.^49^ according to

3 $$u_{1}=\frac{m_{1}}{m_{0}}$$

where *u*_1_ [T] is the normalized temporal moment indicating the average tracer arrival time and *m*_0_ and *m*_1_ are the zeroth and first temporal moments, which were calculated from eq 4 as follows

4 $$m_{j}=\int_{0}^{\infty }t^{j}fdt$$

where *m*_*j*_ is the *j*th temporal moment and *f* is the mass transported at time *t*. Eq 4 was approximated using discrete data as follows:

5 $$m_{j}\approx \overset{n}{\underset{i=1}{\sum }}{\overset{®}{t_{i}\;}}^{j}f\Delta t$$

Here, *i* indicates the sample number. *f* was replaced by *f* = *cQ*, with Q=VΔt and *V* being the volume collected after a change in time Δ*t*. *c* is the concentration of tracer. This allowed estimation of the transported mass in each period. In effect, eq 5 was modified as follows:

6 $$m_{j}\approx \overset{n}{\underset{i=1}{\sum }}{\overset{®}{t_{i}\;}}^{j}cV$$

The time *t* was centered to the timespan in which the sample was collected, where $\overset{®}{t_{i}\;}=\frac{1}{2}\left(t_{i-1}+t_{i}\right)$ . The average transport velocity of tracers (*v*) was determined by dividing the length (*L*) of the soil column over the lysimeter by the average tracer arrival time as follows:

7 $$v=\frac{L}{u_{1}}$$

These parameters allowed a quantitative comparison of phage migration between the land uses and the impact of fluid flow and transport without the requirement to explicate specific interaction mechanisms.^49^

## Results

### Irrigation-Induced Phage Migration and Fluid Flow

The transport and changes in infectivity of tracer phages under field conditions were assessed by quantifying PFUs, tracer phage tCN, and tracer phage vlpCN. Tracer phages were detected in collected samples within minutes after application. This is particularly evident in the breakthrough curves of the lysimeters at the forest sites F1 and F2 and the pasture site P2, where phages appeared almost instantly after application (Figures 1 and S4). Such rapid transport was most reflected in F2, where 90% of all transported phages had passed through the lysimeter 8 h after application (Figure 2A). This is in line with the highest observed average transport velocity of 3–4.1 cm/h, which was more than 3-fold faster than phage transport in F1, P1, and P2 (Figure 2B). In contrast, 90% of the deuterated water was exported after 70 h in F2 with an average transport velocity of 0.68 ± 0.004 cm/h, which was an order of magnitude slower than the transport of the tracer phages. In F1, 50% of phages were transported within 40 h at an average velocity of 0.64–0.77 cm/h, which was almost ten times slower than transport in F2 (Figure 2B). Nevertheless, the phage transport velocity in F1 was twice that of the deuterated water average velocity (0.31 ± 0.003 cm/h), indicating that phages were transported faster than deuterium in F1. Phage transport velocities at the pasture site ranged from 0.66 to 0.83 cm/h in P1 and from 1.6 to 1.64 cm/h in P2, showing distinct transport behavior similar to that observed in the forest sites. At the same time, 90% of the deuterated water mass was transported after 40 h in P2 (Figure 2B) at an average velocity of 1.57 ± 0.02 cm/h, which was slower than the phage transport velocity. Similarly, deuterated water was migrating slower than phages in P1, with an average velocity of 0.56 ± 0.01 cm/h. Irrespective of the land use and enumeration method, tracer phage transport velocity was faster than that of the host fluid, as seen in the later appearance of deuterium, especially in forest sites.

**Figure 1:**
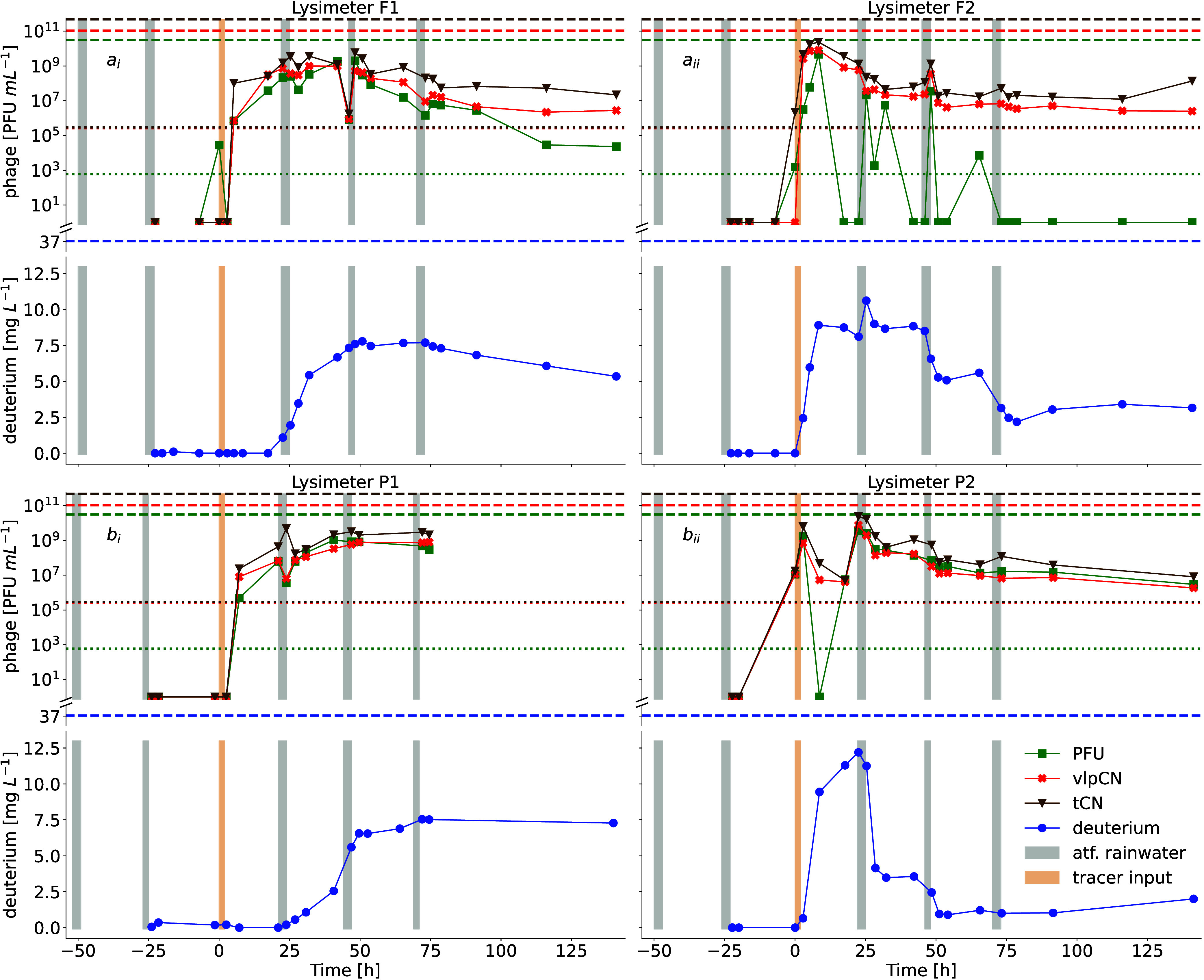
Breakthrough curves of tracer phages and deuterated water (deuterium) under field conditions during the induced transport period. The breakthrough of infectious tracer phages (PFU), DNA from intact tracer phage particles (vlpCN), and total tracer phage DNA (tCN) quantified in the seepage water of lysimeters placed in a forest (a) and pasture (b) site in the Hainich CZO. The panels show each phage quantification (PFU = green squares, vlpCN = red crosses, and tCN = brown triangles), deuterium concentrations (blue cycles), applied tracer concentrations (dashed lines), and limits of quantification of the respective quantification approaches (colored dotted lines). Before and after the application of 4.7 × 10^14^ PFU (equal to 1.61 × 10^15^ vlpCN and 7.18 × 10^15^ tCN) (yellow bar) for 3 h, lysimeters were irrigated with artificial rainwater (gray bars).

**Figure 2:**
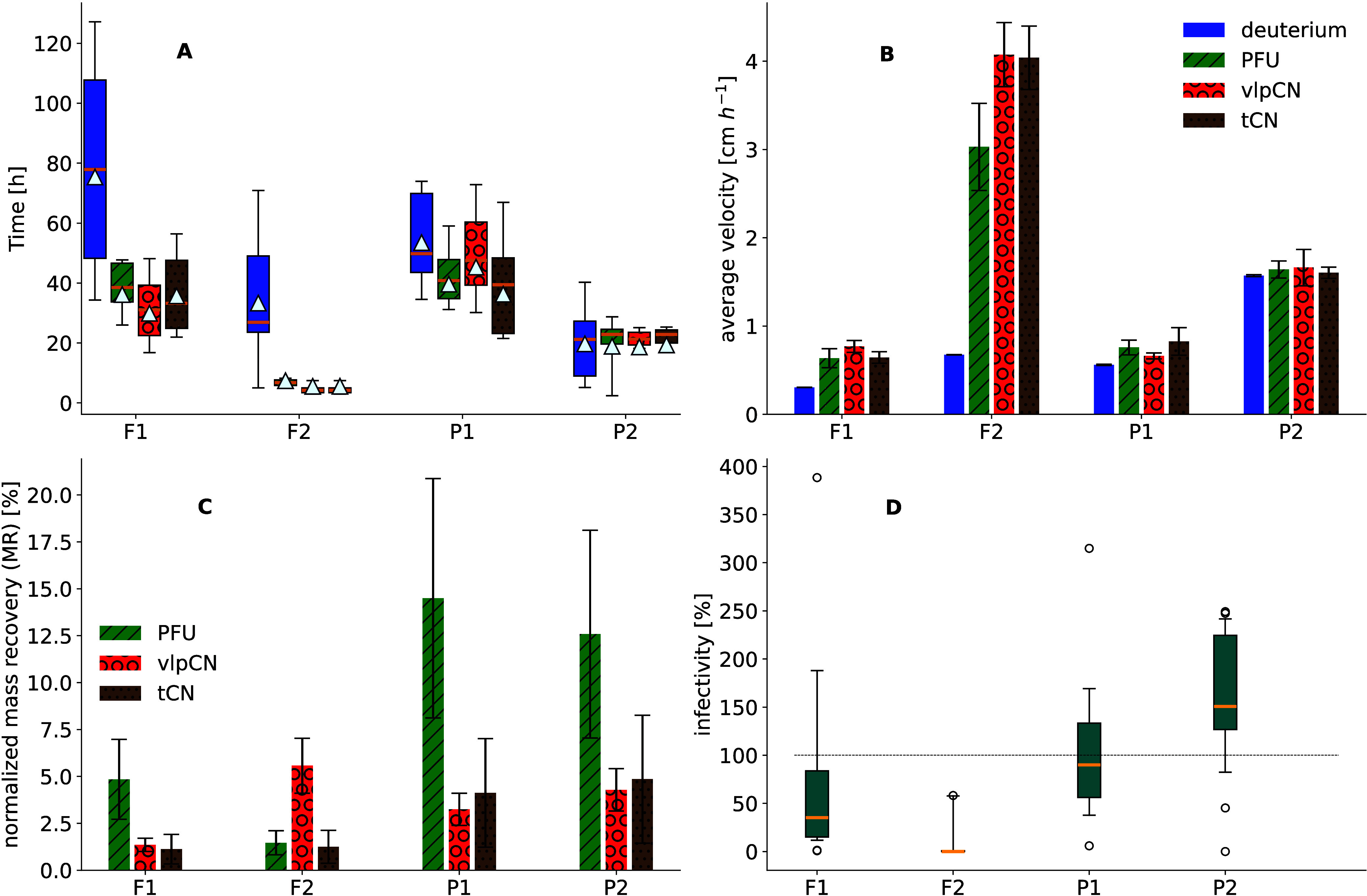
Parameters of tracer phage and deuterated water (deuterium) breakthrough curves under field conditions during induced transport. (A) Relative mass transport of deuterium (blue box), infectious phages (PFU: green box with diagonal line patterns), phage particles (vlpCN: red box with circle patterns), and phage DNA (tCN: brown box with dotted patterns). Whiskers and yellow horizontal lines represent the time it took for 10%, 90%, and 50% of the tracer mass to be transported out of the soil, respectively. The average tracer arrival time (center of mass) is indicated by white triangles. (B) Calculated average transport velocities of deuterium and phage tracers are presented. (C) Recovered phage mass was normalized with total deuterium mass in each lysimeter. Error bars represent the standard error of technical replicates *n* ≥ 2. (D) Changes in specific infectivity during induced transport were observed for all lysimeters. Here, the dashed line represents no loss of infectivity during transport, and whiskers represent the 10th and 90th percentiles of infectivity.

The normalized mass recoveries (MR) differed between enumeration methods and lysimeters and were, in general, higher for PFU than tCN (Figure 2C). For instance, while MR_PFU_ reached 15% in pasture sites, MR_tCN_ was three times lower. A similar pattern was observed in F1, where only 5% of the applied PFUs were transported through the lysimeter. F2 exhibited an even higher loss of PFUs during transport, with MR_PFU_ = 1%, but in parallel, F2 also showed the highest mass transport of vlpCN. Such differences in the loss of PFUs across lysimeter locations were also observed at a later stage of the breakthrough curves. Specifically, 72 h after tracer application, there was still tailing of vlpCN and tCN in all lysimeters, while PFU concentrations differed across lysimeters. While F2 showed a complete loss of infectivity and PFU abundances were significantly lower than vlpCN and tCN numbers in F1 (Figure 1), tailing of PFUs at the pasture site was comparable to vlpCN and tCN. Such site-specific loss of infectivity during transport was also reflected in the PFU-to-vlpCN ratio throughout the induced transport experiment (Figure 2D). Here, F2 showed the highest mean loss of infectivity during transport, with only 8 ± 4% (SE) of vlpCN retaining their infectivity when transported through soil. In addition, only half of the transported phages stayed infective in F1. Conversely, all phages transported in pasture soil remained infective during transport, even 120 h after application. While infectivity loss differed between land covers, the integrity of tracer phage capsids did not show such a trend (Figure S3). Here, the mean vlpCN to tCN ratios of lysimeters varied from 25% to 30% during the induced transport experiment, indicating that infectivity loss is independent of capsid disassembly.

### Precipitation-Induced Phage Migration and Fluid Flow

A pronounced tailing of phage and deuterated water breakthrough was observed in the lysimeters also during the precipitation-induced period (Figure 3), with sampling durations varying among treatments due to sample availability. This long tailing goes in line with phage MR less than 20% during the irrigation-induced period (Figure 2C). Tracer phage tCN and vlpCN could be observed even eight months after tracer application in F2 and seven months after application in P1. In addition, PFUs were still transported 10 months after irrigation in P1. Even if such concentrations were below the limit of quantification (LOQ), clear lysis appeared in the respective plaque assays indicating the presence of infectious phages in the seepage above the limit of detection (LOD). In contrast, the long-tailing of vlpCN and tCN in F2 was not accompanied by the transport of PFUs, and a similar pattern was also observed for F1. Nevertheless, PFUs were detected again in F2 six months after irrigation, indicating survival and remobilization of a small fraction of the applied tracer phages. Such remobilization of PFUs was also observed in P2, coinciding with changes in deuterium concentrations during events of high precipitation.

**Figure 3:**
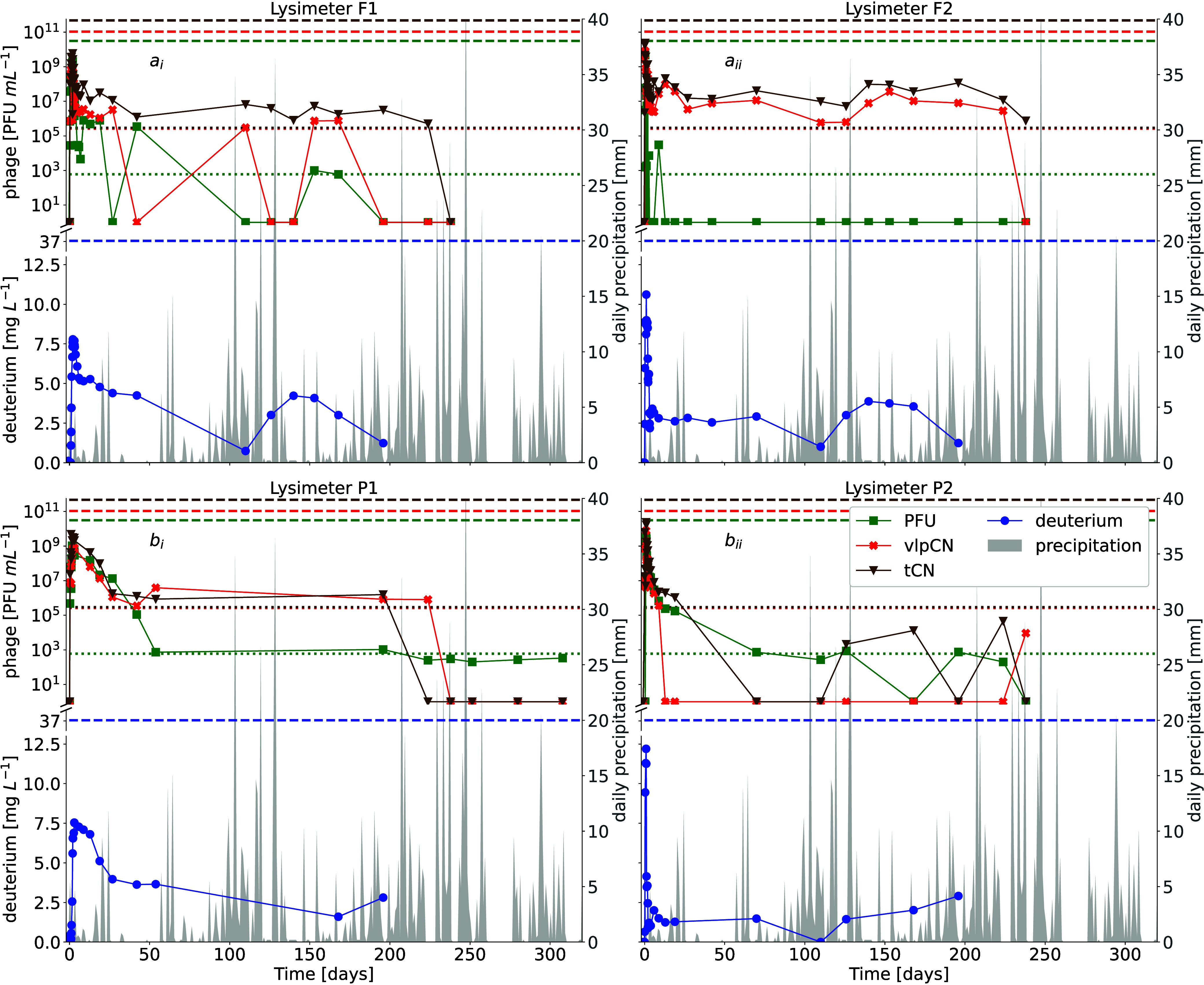
Breakthrough curves of tracer phages and deuterated water (deuterium) under field conditions during the precipitation induced period. The breakthrough of infectious phages (PFU), particle-bound phage DNA (vlpCN), and phage DNA (tCN) of marine tracer *Pseudoalteromonas* phage PSA-HS2 was quantified for about one year in the seepage water of lysimeters placed in a forest (a) and pasture (b) site in the Hainich CZO. The panels show each phage counts (PFU = green squares, vlpCN = red crosses, and tCN = brown triangles), deuterium concentrations (blue circles), applied tracer concentrations (dashed lines), and limits of quantification (dotted lines). In parallel natural precipitation (gray) was assessed.

## Discussion

### Marine Tracer Phages Migrate Faster than Water Independent of Land Cover

To quantify phage transport in undisturbed soil, we compared the movement of tracer phages and deuterated water in soil lysimeters located in areas with different land covers. Although previous studies of nontailed phage transport (e.g., MS2) as surrogates for human viruses are well-documented, long-term studies focusing on the transport dynamics of tailed phages, which are predominantly found in soil ecosystems,^3,4^ are scarce. Our study represents the first long-term assessment of the transport of tailed phages in natural soils under field conditions. Independent of the lysimeter location, tracer phages were transported rapidly and faster than the simultaneously applied deuterated water. This was in line with previous findings in column experiments.^31^ While deuterated water spreads across the entire pore network, including micro- and mesopores, phages with a size of 210 nm^43^ are excluded from pores roughly smaller than 1.5 times their size,^50^ resulting in a substantial pore-size exclusion.^51,52^ Since the mean flow velocity increases when pores accessible to colloids are omitted, phages were transported with a faster velocity, causing them to arrive earlier than deuterated water; an effect also observed under saturated conditions.^53^ In addition, the exclusion from small pores resulted in less dispersed transport of phage mass and a less pronounced tailing effect compared to deuterated water. This is consistent with the study by James and Chrysikopoulos,^54^ who observed that pore-size exclusion reduced the dispersion coefficient. Notwithstanding overall fast transport of HS2, the flow regimes across the study sites were heterogeneous,^8^ which is also reflected by different transport behaviors at different sites for different tracers. The variation in transport velocities between lysimeters is likely due to the heterogeneous pore space. Particularly, the large difference in velocity between phages and deuterated water suggested a higher proportion of preferential flow paths in F2 compared to F1, which was located right next to F2, but with almost an order of magnitude lower transport velocity. Similarly, phage and deuterated water transport velocities in P2 were about twice as fast as those in the nearby lysimeter P1, indicating distinct proportions of larger pores.^12,55^ These findings suggest that distinct phage transport is primarily influenced by soil hydraulic properties rather than the land cover. Additionally, the difference in transport behavior between tracer phages and deuterated water highlights that the conservative tracers are not suited to assess crucial transport properties, such as first arrival, mean velocity, and retention, of (bio)­colloids such as phages in the soil.

### Tailed Phages Exhibit Long-Term Infectivity in Soil and Maintain Their Infectivity Depending on the Soil Type

Infectious phages were found in the outflow of lysimeters nearly one year after application, although marine tracer phages are unlikely to encounter their hosts in soil environments and are therefore presumed to be unable to replicate. Consequently, our long-term detection indicates a high persistence of infectivity in soil. Differences in phage survival were observed between different lysimeter locations. While tracer PFUs were continuously measured in the outflow of pasture lysimeters, forest soil showed reduced transport of infectious phages, even if vlpCN was still eluted in high concentrations. Such long-tailing of vlpCN points at phage adsorption to, e.g., mineral surfaces like clays, followed by desorption.^56–58^ This transport behavior can be attributed to attachment resulting from electrostatic and hydrophobic interactions.^59^

Attachment and subsequent detachment processes have been identified as major drivers of phage infectivity loss,^60–62^ in particular for marine tracer phages.^33^ At the forest site, enhanced phage attachment is likely a result of several factors. In general, forest soil contained more clay,^8^ which is known to be a strong adsorbent for phages.^63^ Due to the smaller grain size of clay compared to, e.g., sand or silt, phage attachment is likely further promoted.^64^ The lower bulk density and higher saturated hydraulic conductivity in forest sites further indicate a less consolidated matrix that permits faster access of fluids to reactive sites. In addition, forest seepage water contained lower concentrations of organic carbon (OC) compared to the pasture site, indicating less parallel transport of phages and OC. As a result, competition between phages and OC for binding sites is likely reduced, facilitating stronger phage attachment during transport.^59^ The expected higher adsorption of phages in forest soil is further consistent with the reduced MR_tCN_ during the induced transport period and the extended tailing of tCN in forest soils in this study. Nevertheless, phage attachment to soil particles can provide protection against infectivity loss, depending on the virus and surface type,^60,65,66^ which complicates the prediction of phage survival in soil.

However, the forest lysimeters showed overall lower pH values (pH 4.8 ± 0.5 and 5.5 ± 0.7) in seepage water,^8^ whereas the pH of pasture seepage (pH 6.3 ± 0.3 and 6.6 ± 0.3)^8^ was within the optimal range for *Pseudoalteromonas* phage infectivity.^67–69^ Seepage water from the forest and pasture sites also differed in their elemental composition.^8^ High copper concentration potentially contributed to the rapid loss of phage infectivity in F2 as already described for the nontailed phage MS2 in batch experiments.^70^ In contrast, Ca and Mg were higher in the pasture seepage and are known in their divalent form to maintain phage infectivity through their specific chemical properties.^71^ As divalent metals, they have been further shown to improve phage infectivity of tailed phages by detangling phage tail fibers and enabling phage adsorption to their host.^19^ The presence of higher Ca and Mg levels in pasture soil could therefore also have contributed to increased phage stability and infectivity compared with forest soil. In particular, tracer phages lost their infectivity almost completely already during the induced transport period in F2 and showed reduced infectivity after transport in F1.

Nevertheless, the remobilization of infectious phages was observed immediately after precipitation events in F1. This remobilization is consistent with the observation of tracer phage retention in the immobile regions in F1. Moreover, such event-driven dynamics have been previously reported for nanoparticles in soil,^8^ transport of topsoil-sourced bacteria during extreme precipitation events,^72^ and remobilization of infectious MS2 phages.^73^ Precipitation events further alter physicochemical soil conditions such as pH and ionic strength, thus likely promoting remobilization of immobilized VLP.^73–75^ This event-driven remobilization is particularly relevant in soil environments, where water connectivity can be limited, allowing particles to accumulate in immobile regions and later be exchanged with the mobile phase during transient flow events.^76^ During these periods of phage immobilization, phage infectivity must be preserved to allow subsequent phage transport to influence the ecological processes. Our study demonstrates that retained phages can remain infectious and be remobilized, supporting recent observations of viral blooms in soil following rewetting events.^21,77^ Although our findings are based only on tracer phages, they highlight potentially important mechanisms in the ecology of soil viruses.

### By Integrating PFU and Genome-Based Phage Counts, the Marine Tracer Phage HS2 Serves As a Promising Tracer for Assessing Biocolloid Transport in Natural Soils

Consistent with previous laboratory column experiments,^31^ the migration of the marine tracer phages differed from that of deuterated water, due to colloidal hydrodynamic effects including pore-size exclusion. However, the combination of dissolved tracers and marine phages allows the delineation of transport processes associated with colloidal hydrodynamics and soil hydraulic properties. To improve the ecological interpretation of tracer studies based on phages, we applied a 3-fold quantification approach involving both counting of PFUs and estimating the amount of tracer phage genomes in intact phage particles (VLPs) or in total DNA. As retained infectious phages can be remobilized, leading to long-tailing and delayed effects, combining both methods offers a more robust understanding of phage transport and its ecological implications. Even when transported PFU concentrations were often below the limit of quantification, such concentrations may still influence microbial communities.^78^ Additionally, marine tracer phages are foreign to the soil system and are assumed not to replicate there. However, autochthonous phages can replicate during transport when encountering their host, potentially amplifying the effects of the water-driven phage movement in soil environments. In addition, since alien tracer phages are potentially less adapted to soil conditions than soil phages,^79^ they can serve as proxies for phage survival but may still underestimate PFU transport and survival of autochthonous phages. Moreover, particle transport in topsoil is reported to be less effective compared to deeper layers,^8,80^ suggesting a potentially even stronger ecological impact of phage transport in the subsoil. However, future studies should investigate how morphological differences in tailed phages influence phage transport and survival in natural soil systems. The ability of phages to persist for extended periods in soil may be crucial for their encounter with susceptible host cells in a highly structured and spatially heterogeneous soil environment. Gaining insights into the environmental factors that shape phage transport and ecology in soil is pivotal for predicting phage impact on nutrient turnover, microbial diversity, and other terrestrial ecosystem services in the soil environment. In summary, marine phages offer promising potential as (bio)­tracers for investigating (bio)­colloid transport, especially the transport of phages and their ecological roles in natural porous media.

## Supplementary Material



## Notes

Additional experimental details including a scheme of the experimental procedure and irrigation parameters as well as extended results (PDF)
