# Supplementary material for: Transport and Survival of Marine Tracer Phages in Topsoil at Field Conditions
Source: Environ Sci Technol. 2025 Dec 23;60(1):677–87. doi: 10.1021/acs.est.5c12252 (PMC12810386; doi:10.1021/acs.est.5c12252)
Supplement: Supplementary file 1 [file es5c12252_si_001.pdf]

Supporting Information

Transport and survival of marine tracer phages in topsoil at field conditions

Konstanze Hild[a, †], Nimo Kwarkye[b,†], Chen Huang[c], Hauke Harms[a,d], Antonis Chatzinotas[a,d,e], Thomas Ritschel[b], Kai U. Totsche[b,f, ‡], Lukas Y. Wick[a, ‡,*]

[a] *Department of Applied Microbial Ecology, Helmholtz Centre for Environmental Research - UFZ, Permoserstraße 15, 04318 Leipzig, Germany.*

[b] *Department of Hydrogeology, Friedrich-Schiller University Jena, Burgweg 11, 07749 Jena, Germany.*

[c] *Department of Biogeochemical Processes, Max Planck Institute for Biogeochemistry, Hans-Knöll-Straße 10, 07745 Jena, Germany.*

[d] *Institute of Biology, Leipzig University, Talstraße 33, 04103 Leipzig, Germany.*

[e] *German Centre for Integrative Biodiversity Research (iDiv) Halle-Jena-Leipzig, Puschstraße 4, 04103 Leipzig, Germany.*

[f] *Cluster of Excellence Balance of the Microverse, Friedrich-Schiller-University Jena, Fürstengraben 1, 07743 Jena, Germany.*

[†]shared first authorship

[‡]shared last authorship

*Corresponding author:

Helmholtz Centre for Environmental Research – UFZ

Department of Applied Microbial Ecology

Permoserstraße 15

04318 Leipzig

Germany

e-mail: lukas.wick@ufz.de

Supplementary Materials:

The file contains 6 pages including Figure S1 to S4 and Table S1 to S6.

1. Extended Materials and Methods

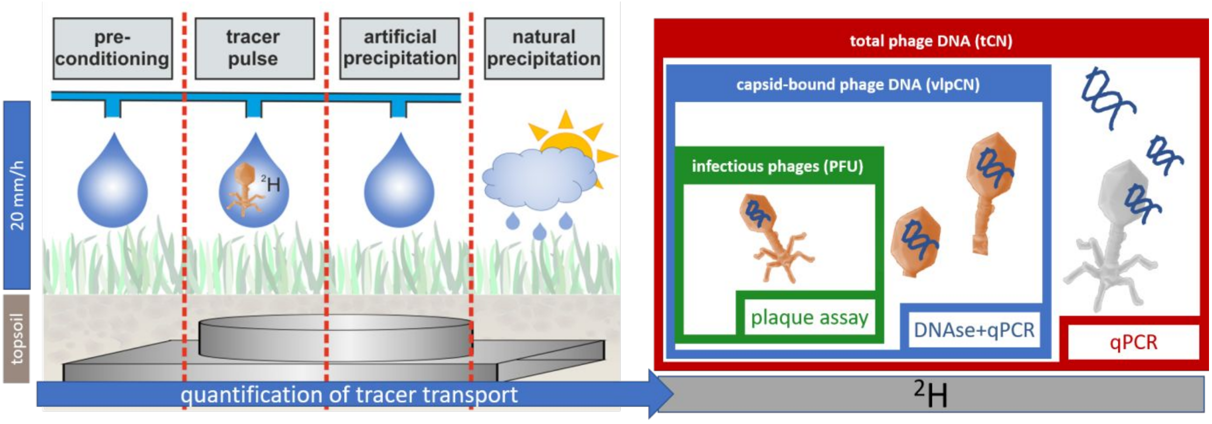

Figure S1. Irrigation scheme and approaches for tracer phage quantification. The breakthrough of deuterium (^{2}H), infectious phages (PFU), particle-bound phage DNA (vlpCN), and phage DNA (tCN) of marine tracer *Pseudoalteromonas* phage PSA-HS2 were quantified in the seepage water of lysimeters placed in a forest and pasture site in the Hainich CZO. Before and after the application of 4.7 x 10^{14} PFU, (equal to 1.6 x 10^{15} vlpCN and 7.2 x 10^{15} tCN) for three hours, lysimeters were irrigated with artificial rainwater. After the induced transport period, water input was supplied by natural precipitation. Seepage water was collected for about one year and phages were quantified by plaque assay and qPCR.

Table S1. Lysimeter depth in forest sites F1 and F2 as well as pasture sites P1 and P2.

Parameter	F1	F2	P1	P2
Depth (cm)	23	22.5	30	31

Table S2. Tracer portfolio with concentration of each tracer applied. PEG represents poly(ethylene glycol).

Applied Tracer	Concentration Applied
Deuterium	200 per mil
Fluorescein	2 mg/L
PEG	50 mg/L
Phage (PFU)	3.1 x 10^{10} PFU/mL
Phage (vlpCN)	1.1 x 10^{11} vlpCN/mL
Phage (tCN)	4.8 x 10^{10} tCN/mL

Table S3. Protocol for applied background irrigation (0.59 mM NaCl) and tracer irrigation at the first forest lysimeter (F1). Uncertainties represent standard errors.

Type	Date	Begin (HH:MM)	End (HH:MM)	Duration (HH:MM)	Volume (L)
Background	17/04/2023	12:55	15:44	02:49	15.5 ± 0.3
Background	18/04/2023	12:51	15:39	02:48	15.4 ± 0.3
Tracer	19/04/2023	14:26	17:10	02:44	15.0 ± 0.0
Background	20/04/2023	12:56	15:44	02:48	15.4 ± 0.3
Background	21/04/2023	12:26	15:14	02:48	15.4 ± 0.3
Background	22/04/2023	12:44	15:32	02:48	15.4 ± 0.3

Table S4. Protocol for applied background irrigation (0.59 mM NaCl) and tracer irrigation at the second forest lysimeter (F2). Uncertainties represent standard errors.

Type	Date	Begin (HH:MM)	End (HH:MM)	Duration (HH:MM)	Volume (L)
Background	17/04/2023	12:58	15:46	02:48	15.4 ± 0.3
Background	18/04/2023	12:53	15:41	02:48	15.4 ± 0.3
Tracer	19/04/2023	14:29	17:13	02:44	15.0 ± 0.0
Background	20/04/2023	12:58	15:46	02:48	15.4 ± 0.3
Background	21/04/2023	12:28	15:16	02:48	15.4 ± 0.3
Background	22/04/2023	12:45	15:33	02:48	15.4 ± 0.3

Table S5. Protocol for applied background irrigation (0.59 mM NaCl) and tracer irrigation at the first pasture lysimeter (P1). Uncertainties represent standard errors.

Type	Date	Begin (HH:MM)	End (HH:MM)	Duration (HH:MM)	Volume (L)
Background	17/04/2023	12:25	15:13	02:48	15.4 ± 0.3
Background	18/04/2023	12:36	15:24	02:48	15.4 ± 0.3
Tracer	19/04/2023	15:29	18:13	02:44	15.0 ± 0.0
Background	20/04/2023	12:41	15:29	02:48	15.4 ± 0.3
Background	21/04/2023	12:14	15:02	02:48	15.4 ± 0.3
Background	22/04/2023	12:35	15:23	02:48	15.4 ± 0.3

Table S6. Protocol for applied background irrigation (0.59 mM NaCl) and tracer irrigation at the second pasture lysimeter (P2). Uncertainties represent standard errors.

Type	Date	Begin (HH:MM)	End (HH:MM)	Duration (HH:MM)	Volume (L)
Background	17/04/2023	12:27	15:15	02:48	15.4 ± 0.3
Background	18/04/2023	12:37	15:25	02:48	15.4 ± 0.3
Tracer	19/04/2023	14:09	16:53	02:44	15.0 ± 0.0
Background	20/04/2023	12:44	15:32	02:48	15.4 ± 0.3
Background	21/04/2023	12:17	15:05	02:48	15.4 ± 0.3
Background	22/04/2023	12:36	15:24	02:48	15.4 ± 0.3

2. Extended Results

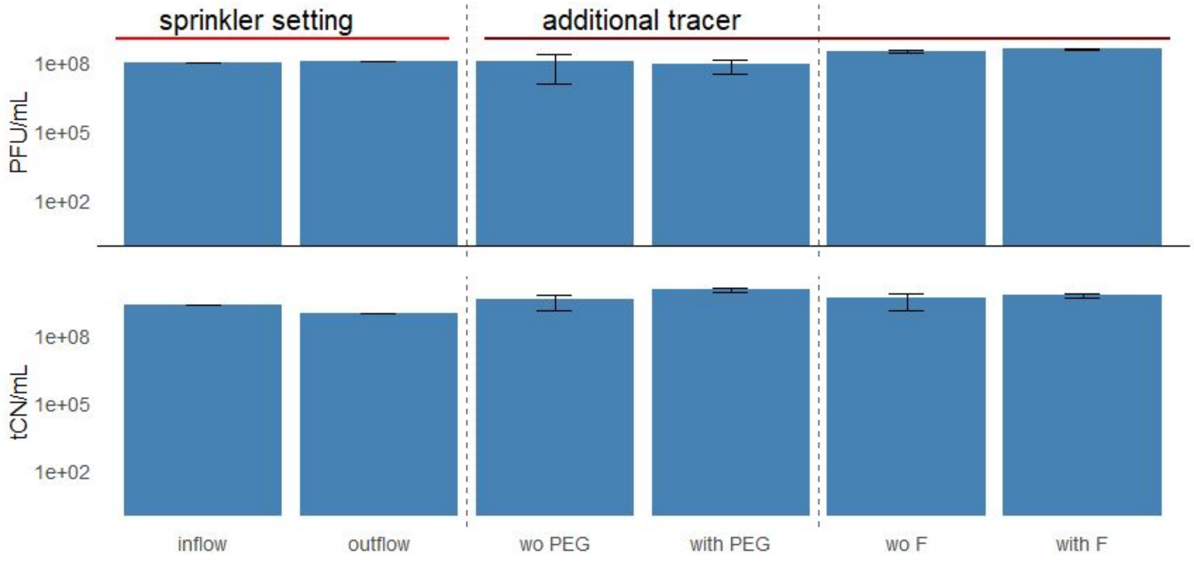

Figure S2. Influence of set-up and additional tracers on tracer phage quantification. Tracer phages were quantified before the application to the sprinkler system (inflow) and after passaging the sprinklers (outflow) (n = 1). In addition, tracer phages were incubated in batch experiments in artificial rainwater with and without (wo) synthesized poly(ethylene glycols) (PEG), or fluorescein (F) (n = 3). Phages were quantified as PFU and tCN. Error bars represent the standard deviation.

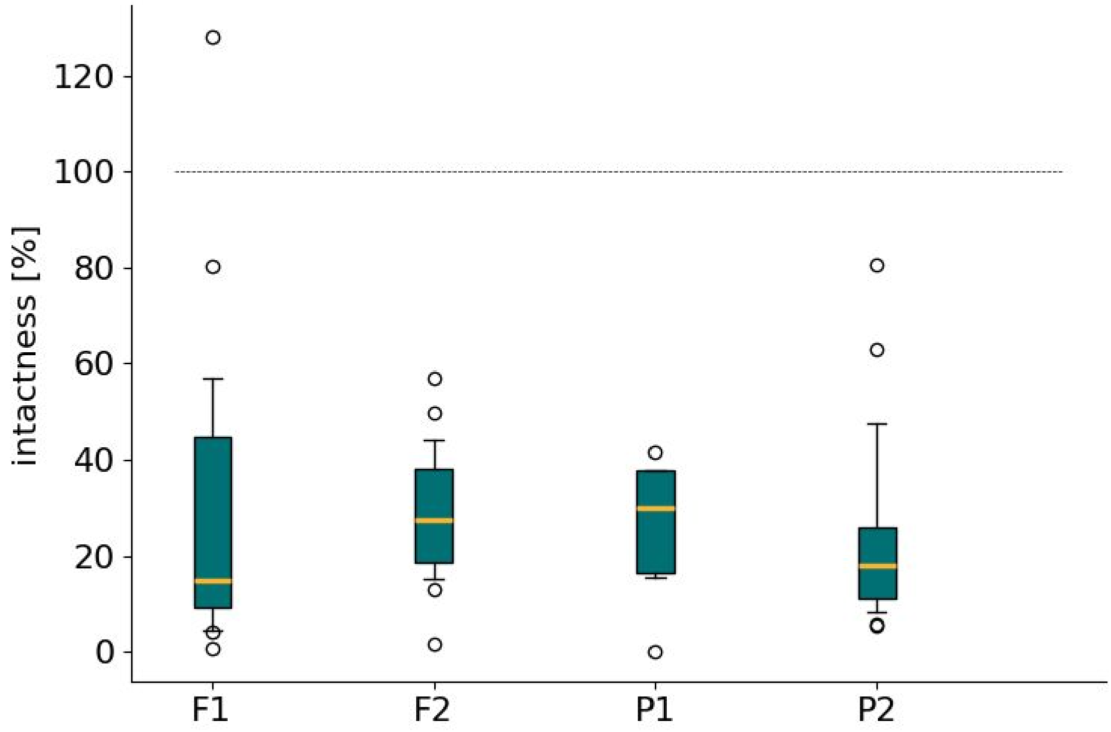

Figure S3. Changes in particle intactness during induced precipitation period across lysimeters. The dashed line represents a scenario where all phage DNA is encapsulated and whiskers represent the 10 and 90 percentile intactness.

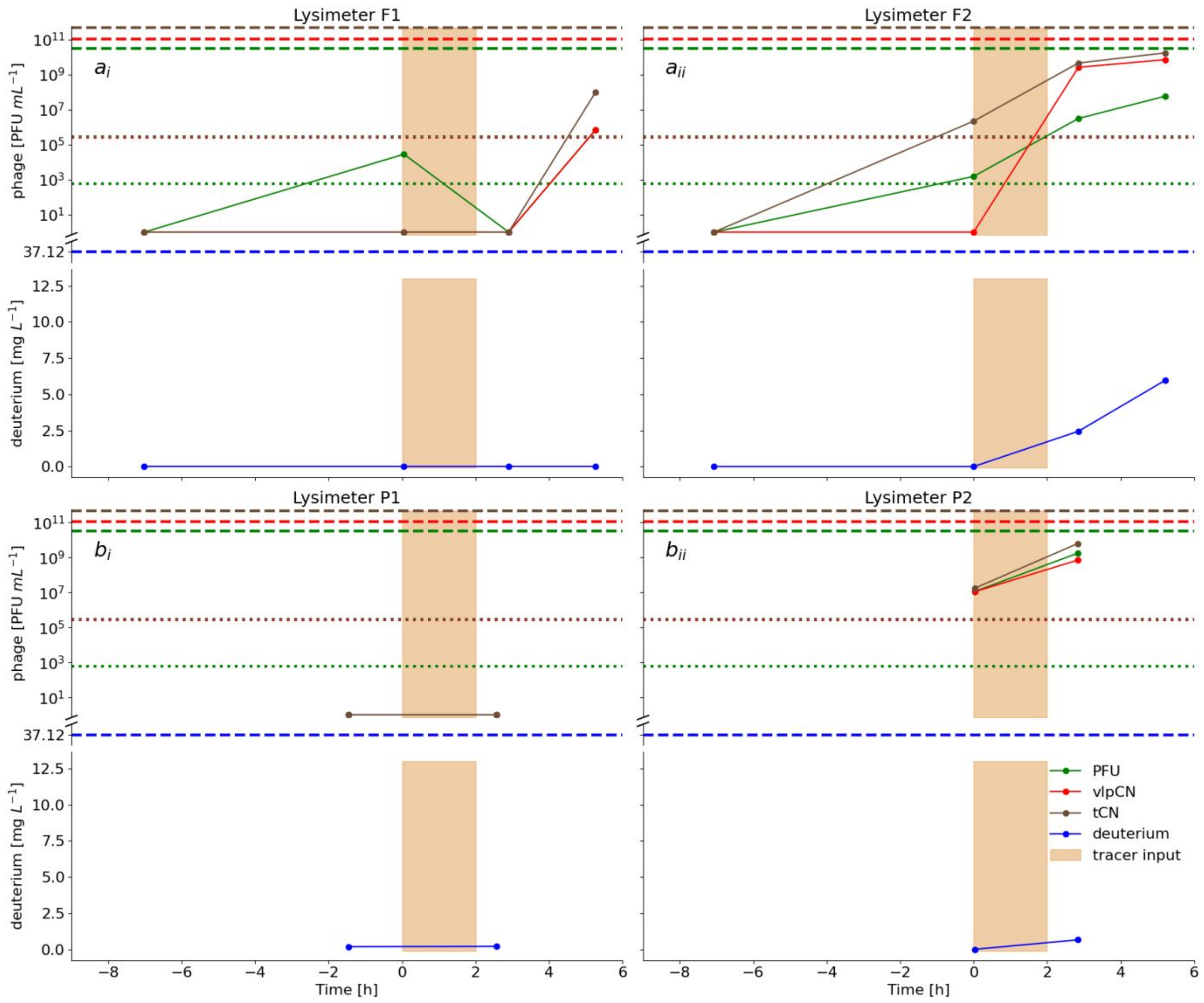

Figure S4: Transport of tracer phages and deuterium in field conditions within 6 hours after tracer application. The breakthrough of infectious tracer phage (PFU), DNA from intact tracer phage particles (vlpCN), and total tracer phage DNA (tCN) quantified in the seepage water of lysimeters placed in a forest (a) and pasture (b) site in the Hainich CZO. The panels show each phage quantification (PFU = green symbols, vlpCN = red and tCN = brown), deuterium concentrations used as a conservative tracer (blue), applied tracer concentrations (dashed lines), and limits of quantification (dotted lines). Before and after the application of 4.7 x 10^{14} PFU, (equal to 1.6 x 10^{15} vlpCN and 7.2 x 10^{15} tCN) (yellow bar) for three hours.
